# Examining the Fraternal Birth Order Effect and Sexual Orientation: Insights from an East European Population

**DOI:** 10.1007/s10508-024-02892-8

**Published:** 2024-06-13

**Authors:** Jakub Fořt, Benjamin Kunc, Jaroslava Varella Valentova, Klára Bártová, Kateřina Hudáčová

**Affiliations:** 1 Department of Zoology, Faculty of Science, Charles University, Viničná 7, 128 00 Prague, Czechia; 2 Department of Psychology, Faculty of Arts, Charles University, Prague, Czechia; 3 Department of Neurosciences, Faculty of Medicine, KU Leuven, Leuven, Belgium; 4 Department of Experimental Psychology, Institute of Psychology, University of Sao Paulo, Sao Paulo, Brazil; 5 Department of Psychology and Life Sciences, Faculty of Humanities, Charles University, Prague, Czechia; 6 National Institute of Mental Health, Klecany, Czechia

**Keywords:** Male homosexuality, Female homosexuality, Sexual orientation, Maternal fertility, Birth order

## Abstract

The development of human sexual orientation remains a complex and multifaceted subject. It is often studied but its origins continue to elude us. In this preregistered study, our primary objective was to demonstrate the fraternal birth order effect (FBOE), which assumes a higher prevalence of older brothers in gay men than in their straight counterparts and which has also been recently recorded in lesbian women. Our second aim was to explore any potential impact of the FBOE on anal-erotic role orientation (AERO), both in gay and straight men. Our study sample included 693 gay men, 843 straight men, 265 lesbian women, and 331 straight women from Czechia and Slovakia. Employing a conventionally parameterized logistic regression model, we substantiated the FBOE among both gay men (OR = 1.35 for maternal older brothers) and lesbian women (OR = 1.71). These outcomes were confirmed by a more nuanced parameterization recently proposed by Blanchard (2022). Nonmaternal older brothers did not exhibit a significant influence on their younger brothers’ sexual orientation. Contrary to some earlier reports, however, our data did not establish the FBOE as exclusive to gay men with the receptive AERO. Furthermore, our observations indicated a lower offspring count for mothers of gay men compared to mothers of straight men. Emphasizing the positive FBOE outcomes, we acknowledge the need for caution regarding the various options that can be used to estimate the familial influences on sexual orientation.

**Supplementary Information:**

The online version contains supplementary material available at 10.1007/s10508-024-02892-8.

## Introduction

Previous research had suggested several proximate factors that influence the development of human sexual orientation, including genes (Ganna et al., 2019; Hamer et al., 1993), epigenetic influences (Rice et al., 2012), hormones, especially prenatal (Tasos, 2022), and an immunological reaction. In this study, we focused on a possible immunological mechanism that has been proposed to explain the observed higher number of older brothers among gay men than in straight men (Blanchard & Klassen, 1997; Bogaert et al., 2018).

### The Fraternal Birth Order Effect

The sibling composition of gay men first attracted the attention of researchers in the 1930s and 1940s (see Lang, 1940). Several decades later, this interest was renewed in the 1990s. Blanchard and Bogaert (1996) examined the number of siblings in their sample of gay and straight men and reported an excess of older brothers in gay men. No differences were found in the numbers of other siblings. In the following years, the proposed fraternal birth order effect (FBOE) found significant further support in other studies conducted in various countries and populations (e.g., Ablaza et al., 2022; Apostolou, 2020; Gómez Jiménez et al., 2020; King et al., 2005; Raymond et al., 2023). Ablaza et al. (2022) recently substantiated the FBOE based on a huge dataset from a Dutch census using a sophisticated method to control for confounding variables but some other studies failed to find support for the effect (see Blanchard, 2018a and Blanchard et al., 2020 for recent meta-analyses and for examples of studies not supporting the effect).

The FBOE in gay men is usually explained by the maternal immune hypothesis (Blanchard & Klassen, 1997), which was proposed as a sex-asymmetrical immunological cause of male homosexuality. It assumes that cellular, protein-containing material from embryos or fetuses of the male sex can permeate the placenta, enter maternal bloodstream, and lead to an antibody-mediated immunological response to male-specific antigens. When the woman is pregnant again, these antibodies cross the placenta, invade the unborn child’s circulation, and alter some of the child’s physiological functions. The levels of maternal antibodies are supposed to increase with each subsequent pregnancy with a male fetus. The validity of the maternal immune hypothesis was partly corroborated by the only direct test to-date (Bogaert et al., 2018), which found that the blood levels of anti-NLGN4Y (neuroligin 4 Y-linked is a male-specific cell-adhesion protein present in neural synapses) antibodies are higher in the mothers of gay sons than in the mothers of straight sons or no sons. Nevertheless, the study did not find a significant difference between the mothers of gay sons with older brothers and mothers of gay sons with no older brothers (for anti-NLGN4Y isoform 1 or significance-bordering results for isoform 2 or isoforms 1 and 2 combined), which presents a challenge for the maternal immune hypothesis (Valentova et al., 2023).

Yet there is another complementary possible explanation (not necessarily contradictory to the MIH), which has not received much theoretical attention so far. As Haig (2014) outlined, there is a potential that older siblings may influence their younger siblings’ physiology through fetal microchimerism. Fetal microchimerism is a naturally occurring state in which fetal cells, after crossing the placenta, engraft into various tissues in the maternal organism (Cómitre-Mariano et al., 2022). In many cases, the physiological function of these engrafted cells of fetal origin is not clear–they may probably serve as a beneficial factor in maternal health but are also believed to contribute to autoimmune diseases (Cómitre-Mariano et al., 2022). In the development of human sexual orientation, cells of older brothers engrafted in maternal tissues might (1) contribute to the continuous production of antibodies against male antigens or, which is highly speculative, (2) even re-cross the placenta, enter the younger siblings’ bloodstream and alter their physiology directly (see also Haig, 2014). Obviously, other ways these cells could exert their influence are conceivable. Still, the cardinal background assumption, i.e., mothers of homosexual men having a higher amount of male microchimerism in comparison with mothers of heterosexual men, remains to be tested empirically.

Whatever the exact mechanism, the available evidence suggests that male homosexuality or a disposition to male homosexuality is at least partly influenced prenatally and that older brothers affect their younger brothers sexual orientation in a biological, nonsocial way. Therefore, we should observe no excess of nonbiological older brothers in gay men–and that is, indeed, the case (Bogaert, 2006). Of note is the pioneering discovery of an FBO-like effect in the first non-human species. Hernández et al. (2024) recorded that males born to multiparous laboratory rats show higher proportions of same-sex partner preference and homosexual behavior than males born to primiparous females. This, if further replicated, could potentially hint at the etiology of same-sex preference that is shared across species.

Wampold (2018b) and Swift-Gallant et al. (2018) contributed a new piece to this puzzle. Wampold (2013) reasoned that the FBOE might be present only in gay men with receptive anal-erotic role orientation (thereinafter AERO). He based his thoughts on studies showing that gender-nonconforming individuals have a higher number of older brothers and that gay men with receptive AERO tend to display feminine personality traits (Zheng et al., 2012). To test this hypothesis, Swift-Gallant et al. (2018) divided their male participants into preferential tops, bottoms, and versatiles (i.e., those who prefer to penetrate their partner anally, those who prefer to be anally penetrated, and those with no preference either way). They found that bottoms significantly diverge from both the general population and from versatiles in having a higher number of older brothers. In contrast, Wampold (2018b) reported a higher number of older brothers only in bottoms who actually behave in this manner; they had a higher number of older brothers than tops did.

Wampold (2018a) had also suggested that the FBOE could be related not to male homosexuality as such but rather to the wish of being anally penetrated, regardless of whether the individual is gay or not. In fact, Wampold et al. (2018) presented preliminary evidence to the effect that sexually submissive straight men from the BDSM community have a higher number of older brothers than the non-submissives. This evidence is, however, limited because general sexual submissiveness is a concept different from receptivity during anal intercourse.

The findings presented above, however tenuous they may be, could imply that there are two or more “kinds” of gay men: bottoms, who display feminine personality traits and the FBOE applies to them, and non-bottoms, in whom we do not find the FBOE. Results reported by Swift-Gallant et al. (2019) and Swift-Gallant et al. (2021) support this position. Swift-Gallant et al. (2019) surveyed gay men and concluded there are at least three distinct groups of gay males according to the three following biomarkers: older brothers, familiality (i.e., having a gay relative), and non-righthandedness. These biomarkers were mutually exclusive in each group, although the differences did not reach the level of formal significance. The analysis has also revealed the largest, fourth group, in whom none of these three biomarkers were present (see also Swift-Gallant et al., 2021 for results on the 2D:4D ratio, a putative marker of prenatal androgenization, and its relation to gay men with different AERO).

Since nearly all previous FBOE studies were conducted without taking the AERO into account, gay men with the top AERO may have obscured the presence of the FBOE in gay men from the bottom group. It is therefore crucial to consider the AERO when studying the FBOE in both gay and straight men. One of the objectives of our study was to explore this possibility by comparing the number of older brothers in straight men who differ with respect to their heterosexual AERO (by investigating whether, when engaging in or fantasizing about straight anal sex, they score as tops or bottoms).

Some studies have moreover reported a higher number of older sisters in gay than in straight men, and this so-called sororal birth order effect (SBOE) was recently confirmed in a meta-analysis by Blanchard et al. (2021). Its explanation, however, is not straightforward. Blanchard and Lippa (2021) point out that there is a correlation between the number of a woman’s liveborn offspring and total miscarried fetuses (Cohain et al., 2017). Blanchard and Lippa (2021, p. 798) conjecture that “mothers who have delivered more daughters are more likely to have ‘seen’ male antigen than are mothers who have delivered fewer daughters, and that the former are therefore slightly more likely than the latter to produce a homosexual son in a subsequent pregnancy.” Moreover, a study of Raymond et al. (2023) suggests that the SBOE can emerge as a mere statistical artifact in samples where the genuine FBOE is present simply because the number of older sisters correlates with the number of older brothers.

Recently, the FBOE has been reported also among lesbian women (Ablaza et al., 2022; but see Blanchard & Skorska, 2022 or Xu & Zheng, 2017 for null findings). This is not easy to explain because the maternal immune hypothesis formulated by Blanchard and Klassen (1997) states that maternal immune system is activated only by proteins originating in male fetuses, which is why the maternal antibodies should affect only male fetuses and male sexual orientation. Some authors speculate that maternal anti-NLGN4Y antibodies could react with NLGN4X, which is an X-chromosome-based homolog of the NLGN4Y protein, in a similar manner and with similar consequences (Blanchard, 2022). These findings highlight the fact that women are all too often overlooked as subjects of research into the FBOE and biological underpinnings of sexual orientation (Blanchard, 2023), which is a grave error if we want to formulate a truly adequate theory of the etiology of human sexual orientation (for a recent review regarding female homosexuality, see Luoto et al., 2019). In the present study, our aim was to contribute to this goal by, among other things, including female subjects.

### Analytic Approaches Toward the Fraternal Birth Order Effect

A possible presence of the FBOE is usually investigated using logistic regression. A typical logistic regression model aimed to test the FBOE is usually parameterized by entering into the model the four main predictors of interest, i.e., the numbers of older brothers, older sisters, younger brothers, and younger sisters, possibly along with some secondary predictors. If any of these predictors crosses the significance criterion, it is interpreted as a predominance of that sibling category in homosexual or heterosexual (or bisexual, mostly straight, etc.) subjects, depending on the value of the odds ratio and the reference level. If the significant predictor is the number of older brothers, the odds ratio is greater than one, and heterosexual subjects constitute the reference level in the case of a binary regression model, it is concluded that homosexual subjects have an excess of older brothers in comparison to heterosexual subjects (see, for example, Apostolou, 2020; Blanchard & Bogaert, 1996; Wampold, 2018b for this type of parameterization). Ablaza et al. (2022) in their article criticized this parameterization for the key predictor of interest (the number of older brothers) not reflecting *only* the number of older brothers. When the number of older brothers is increased by one, the total amount of older siblings (irrespective of their sex) and total sibship size also increase by one. This poses a challenge for interpreting potential positive results, especially given that there is some evidence to the effect that gay men’s mothers are more fertile than straight men’s mothers (see, e.g., Camperio Ciani & Pellizzari, 2012, for theoretical background), and gay men may thus potentially have more siblings. Ablaza et al. (2022) therefore proposed a novel and more valid parameterization that tackles these confounding factors. Instead of the numbers of the four categories of siblings, the four key predictors are (1) the total number of siblings (sibship size), (2) the number of older brothers, (3) the number of younger brothers, and (4) the number of older siblings of either sex. Ablaza et al.’s (2022) novel parameterization has already been welcomed and praised by other researchers in the field (Blanchard, 2022; Semenyna et al., 2022). In the following, we will therefore employ both analytical approaches to compare possible differences in the results which they deliver.

### The Aims of the Current Study

The main aim of this study was (1) to verify the presence of the FBOE among gay men. Further, we tested whether (2) the FBOE is restricted to gay men with the bottom AERO, (3) the FBOE is present in straight men with the bottom straight AERO, and (4) the FBOE is present also in women. We test these aims on a previously unstudied Czech and Slovak population and compare different analytical approaches.

## Method

### Participants

We have collected data via an online questionnaire designed in Qualtrics. The survey session was started by a total of 3963 respondents. We excluded (1) participants who did not indicate their sex (*N* = 243). Additional respondents were excluded, in accordance with the preregistration of our study, if they (2) were less than 18 years old (*N* = 42), (3) indicated their sex as “other” (*N* = 50), considered themselves (4) asexual (*N* = 111), (5) transgender (*N* = 119), (6) were classified as bisexuals (*N* = 520), (7) reported being adopted (*N* = 63), or (8) if they failed to fill in all five items to classify their sexual orientation (*N* = 820; see Supplementary Figure S1). For a complete report of the outlier elimination and data cleaning procedures, see Supplementary methods. Some potential participants met multiple exclusion criteria. Data collection spanned between June 2020 and March 2021. The final male sample included 843 straight (*M* = 32.89 years, *SD* = 11.95) and 698 gay (*M* = 32.62 years, *SD* = 11.21) men as based on their mean score on the five Kinsey scale items (see Supplementary Figure S1 and Analyses). The final female sample consisted of 596 women, 331 of whom were classified as straight (*M* = 31.77 years, *SD* = 9.78) and 265 as lesbian (*M* = 27.75 years, *SD* = 6.71).

### Procedure

The online questionnaire was distributed in Czech to both Czech and Slovak participants. Czech and Slovak are very similar languages and Slovaks tend to understand Czech perfectly and vice versa. The questionnaire contained a brief introduction including a consent form, which described the general purpose of the study and reminded respondents that they have the right to terminate their participation and withdraw from the study at any time. The introduction was followed by two sections of items relevant to this research (and two additional sections not relevant to this research, see Measures).

To ensure a sufficient similarity between the homosexual and heterosexual part of our sample with respect to sociocultural and religious factors, we used the targeted sampling method previously employed by Camperio Ciani et al. (2004) and Iemmola and Camperio Ciani (2009). First, we asked various Czech and Slovak LGBT+ friendly organizations to share the questionnaire on their platforms, which were mainly Facebook and web pages. Some organizations also disseminated the questionnaire among friends and acquaintances of their members. Subsequently, we contacted organizations not associated with the LGBT+ community but resembling the previously contacted organizations in their sphere of interest. This was, of course, possible only after a subsample of homosexual individuals was collected (for details, see the list of these organizations in the Acknowledgements). Additionally, we employed targeted advertising on Facebook to gather more data from straight and gay men. The audience we targeted and were trying to reach were men 20–65 years old, living in Slovakia or Czechia, whose Facebook interests (only in the gay subsample) matched “gay pride,” “homosexuality,” or “LGBT community.”

### Measures

The questionnaire included two sections relevant for this specific study. Demographic characteristics included age, sex (with options “male,” “female,” and “other”), the number of own biological daughters and sons, the number of older and younger full-, half-, and stepbrothers and sisters (each response on a slider from zero to ten and more, plus an “I don’t know” option), marital status, education, occupation, the importance of religious faith (from 0 corresponding to “not important” to 100 being “very important”), population size of the childhood place of residence, population size of the current place of residence, an item to determine whether the respondent was adopted, and mother’s age. Population sizes of the current place of residence and in childhood were indicated on a 5-point ordinal scale (with options: less than 1000 inhabitants, 1000–5000 inhabitants, 5000–50,000 inhabitants, 50,000–500,000 inhabitants, and over 500,000 inhabitants).

Sexuality-related questions included the Kinsey scale of self-identification of sexual orientation and four related items regarding sexual fantasies, desire, and the sex of sexual partners during the past year and in the last five years (all 7-point scales, see Supplementary Figure S1). The same items had been used by Iemmola and Camperio Ciani (2009) and Camperio Ciani et al. (2009). This section also included items used to classify the respondents’ anal-erotic role orientation behavior (AERO behavior) during same-sex and opposite-sex anal intercourse: “During heterosexual anal intercourse” and “During homosexual anal intercourse,” with options “I always take the active position, I penetrate my partner,” “I mostly take the active position,” “I take the active and passive position about equally often,” “I mostly take the passive position,” “I always take the passive position, I am penetrated by my partner,” and “I do not engage in such activities.”

During the study, a second pair of items was added to ascertain participant’s AERO preferences, namely “Which position do you *prefer* to hold when engaging in same-sex anal intercourse or when imagining it?” plus an analogous item for opposite-sex anal intercourse. These items had options worded similarly as in the previous item. Swift-Gallant et al. (2018) and Wampold (2018b) had asked their participants technically the same two pairs of questions (regarding AERO behavior and AERO preferences), but the wording of the items somewhat differed between the two studies, and it slightly differs from the wording of our items as well.

This section also included two items investigating whether the respondent is asexual or transgender and the total number of times the respondent had ever engaged in same-sex or opposite-sex anal intercourse. The wording of this section was different for men and women: the terms “men/man” and “women/woman” were altered depending on the sex of the participant. See our preregistration for details: https://osf.io/ugx6t/.

### Statistical Analyses

The data were prepared in Excel and analyzed in Jamovi (The Jamovi Project, 2022) and RStudio (Posit Team, 2023). To classify respondents based on their sexual orientation, we computed the mean score of five sexuality-related items (Figure S1, see Camperio Ciani et al., 2009 and Iemmola & Camperio Ciani, 2009 for the same procedure). Respondents with a mean score 0–1 (inclusive) were labeled as straight, respondents with a mean score 5–6 (inclusive) were classified as gay/lesbian, and those with a mean score 1–5 (exclusive) were labeled as bisexual and excluded from the study. Maternal siblings (older brothers, younger brothers, older sisters, and younger sisters) were calculated as the sum of siblings and half-siblings with whom the participant shared the mother, while nonmaternal siblings were calculated as the sum of stepsiblings and half-siblings with a shared father. Participant’s total sibship size was calculated as the sum of all maternal siblings (participant not included). Mother’s age at childbirth was calculated by subtracting the participant’s age from the mother’s age. Missing values in this variable were substituted with arithmetic means separately for gay men, lesbian women, straight men, and straight women.

AERO behavior: for gay men, those who indicated “I always hold the active position” or “I mostly hold the active position” and had engaged in gay anal intercourse at least 30 times (to assure sufficient experience with anal sex, see Wampold, 2018a for applying the same criterion) were labeled as tops, while those who indicated “I mostly hold the passive position” or “I always hold the passive position” and indicated they had engaged in anal intercourse at least 30 times were labeled bottoms. Versatiles were not analyzed. For straight men, those who indicated “I always take the active position” or “I mostly take the active position” and had engaged in straight anal intercourse at least three times were labeled tops, while those who indicated “I take active and passive positions about equally,” “I mostly take the passive position,” or “I always take the passive position,” and had engaged in anal intercourse at least three times were labeled bottoms. Here, we included straight versatiles in the bottom group and lowered the required number of times of anal intercourse to three in order to increase the sample size. In this way, instead of just four straight men who always or mostly take the passive position and had anal intercourse at least 30 times, we were able to increase the sample size to 33 straight men who always or mostly take the passive position or are versatile and had anal intercourse at least three times. Even so, it is a regrettably low number. AERO preferences categories were calculated essentially in the same way except that there was no requirement for any instances of previous anal intercourse.

We have also computed the older brothers’ odds ratio (OBOR; Blanchard, 2018b) for all analyses of maternal older brothers’ influence on sexual orientation or the AERO. The OBOR was computed as:

(homosexuals’ older brothers/homosexuals’ other siblings)/(heterosexuals’ older brothers/heterosexuals’ other siblings),

where other siblings = older sisters + younger brothers + younger sisters.

In a similar way, OBOR was computed for AERO, with the terms being bottom gay men’s siblings and top gay men’s siblings. OBORs were calculated online using https://www.medcalc.org/calc/odds_ratio.php.

According to our preregistration, for each regression model we have originally tested whether the groups (e.g., gay and straight men, top and bottom gay men, etc.) significantly differ in any of the following variables: age, the importance of religious faith, and the population size of the childhood place of residence and of the current place of residence. When we found a significant difference, we added the relevant variable as a covariate in a specific analysis. If, for instance, we were to find a difference in the importance of religious faith between straight and lesbian women, we would enter this variable as a covariate in all analyses considering lesbian and straight women.

Nonetheless, as suggested during the peer review process, this procedure to control for potential confounds may be erroneous because the addition of religiosity and population sizes as covariates may absorb a variation that should be attributed to focal sibling characteristics. We have therefore decided to control only for participants’ age and age of their mothers at childbirth. Inclusion of these (and only these) covariates also increased the comparability of our analyses with the results of previous studies that employed a similar procedure in controlling for confounds (e.g., Ablaza et al., 2022; Blanchard & Bogaert, 1996; Bogaert, 2006). The regression models presented and discussed in this article therefore include only participants’ age and their mothers’ age at childbirth as covariates. Models which control for confounds in a way described in our preregistration and in the previous paragraph can be found in Supplementary material (Tables S1–S12).

We used binary logistic regression models and Wilcoxon signed-rank tests for paired data. All alpha significance levels for confirmatory testing of hypotheses (i.e., testing hypotheses on the male sample) were set to 0.01 to reduce Type I error rate due to multiple hypotheses testing and to prevent Type II error that would be unacceptably high if a more conservative correction was used (see Nakagawa, 2004). In the exploratory part, where women were the prime focus, we used the standard 0.05 significance criterion. The significance level for testing between-group differences in demographic variables was set to 0.05. 

When we were preparing our preregistration, i.e., during the initial planning of the project and data analysis, Ablaza et al. (2022) had not yet published their novel parameterization of the regression model (see Introduction). By the time the novel parameterization had appeared, we had already collected and analyzed our data. We decided to present the results we have already had and, additionally, to reanalyze all relevant hypotheses using the novel parameterization proposed by Ablaza et al. (2022).

## Results

Descriptive statistics of the male sample are summarized in Tables 1 and 2. To explore the differences in sociodemographic variables between gay and straight males, we used Welch’s *t*-tests for continuous variables and chi-squared tests for categorical variables. Variables with statistically significant differences were the population size of the place of residence in childhood (χ^2^ = 17.37, *p* = 0.002) and at present (χ^2^ = 16.18, *p* = 0.003), and the importance of religious faith (*t* = 5.61, *p* < 0.001; see Tables 1 and 2 for details). There was no significant difference in the participants’ age (Table 1).

**Table 1:** Descriptive statistics for age and religiousness in the male sample

	Sexual orientation	*N*	Missing	*M*	*SD*	Median	*p*
Age	Straight	843	0	32.89	11.95	30.00	.648
	Gay	698	0	32.62	11.21	30.00	
Importance of religious faith (0–100)^a^	Straight	751	92	30.93	33.84	16.00	< .001
	Gay	610	88	21.39	28.90	10.00	

^a^Where 0 means “not important” and 100 “very important”

**Table 2:** Descriptive statistics for education and population sizes of places of residence in the male sample

	Level	Sexual orientation				Total	*p*
		Straight		Gay			
		*N*	%	*N*	%		
Education	Primary	33	3.9	27	3.9	60	
	Upper secondary (vocational)	34	4.0	47	6.7	81	
	Upper secondary (high school graduation)	348	41.3	276	39.5	624	
	Tertiary (bachelor’s degree or equivalent)	121	14.4	93	13.3	214	.216
	Tertiary (master’s degree or equivalent)	247	29.3	214	30.7	461	
	Tertiary (doctorate or equivalent)	55	6.5	37	5.3	92	
	< missing >	5	0.6	4	0.6	9	
Population size of place of residence in childhood	< 1000	83	9.8	109	15.6	192	
	1000–5000	151	17.9	119	17.0	270	
	5000–50,000	247	29.3	223	31.9	470	.002
	50,000–500,000	234	27.8	151	21.6	385	
	> 500,000	125	14.8	95	13.6	220	
	< missing >	3	0.4	1	0.1	4	
Population size of place of residence current	< 1000	72	8.5	66	9.5	138	
	1000–5000	108	12.8	68	9.7	176	
	5000–50,000	161	19.1	106	15.2	267	.003
	50,000–500,000	258	30.6	204	29.2	462	
	> 500,000	224	26.6	245	35.1	469	
	< missing >	20	2.4	9	1.3	29	
Total		843		698		1541	

### The Fraternal Birth Order Effect in Men

Descriptive statistics regarding the number of siblings can be seen in Table 3. Table 4 shows the results of the conventionally parameterized model (all VIFs < 2). As predicted, this model showed that the number of maternal older brothers had significantly increased the log odds ratio that the participant is gay (*b* = 0.30, *p* < 0.001, OR = 1.35 [95% CI: 1.14, 1.60]). Not predicted was the significant negative effect for the number of maternal younger sisters which we found (*b* = -0.39, *p* < 0.001, OR = 0.67 [95% CI: 0.56, 0.82]). In line with our expectations, we found no effect for nonmaternal older brothers (*b* = 0.07, *p* = 0.693, OR = 1.07 [95% CI: 0.77, 1.48]). OBOR statistic for older brothers yielded a significant result (*z* = 4.55, *p* < 0.001, OBOR = 1.58 [95% CI: 1.30, 1.92]).

**Table 3:** Descriptive statistics of siblings of straight and gay men

	Sexual orientation					
	Straight			Gay		
	*N*^a^	*M*	*SD*	*N*^a^	*M*	*SD*
Maternal younger brothers	843	0.43	0.71	698	0.35	0.66
Maternal older brothers	843	0.30	0.63	698	0.41	0.68
Maternal younger sisters	843	0.38	0.62	698	0.26	0.55
Maternal older sisters	843	0.31	0.63	698	0.34	0.67
Nonmaternal younger brothers	843	0.11	0.46	698	0.11	0.42
Nonmaternal older brothers	843	0.07	0.32	698	0.08	0.34
Nonmaternal younger sisters	843	0.09	0.35	698	0.07	0.30
Nonmaternal older sisters	843	0.07	0.29	698	0.10	0.35

^a^There were no missing values in any of the variables

**Table 4:** Fraternal birth order effect in men

						95% CI	
	*b*	*SE*	*Z*	*p*	OR	Lower	Upper
Intercept	1.77	0.42	4.16	< .001	5.86	2.55	13.47
Maternal younger brothers	− 0.20	0.08	− 2.43	.015	0.82	0.70	0.96
Maternal younger sisters	− 0.39	0.10	− 4.07	< .001	0.67	0.56	0.82
Maternal older brothers	0.30	0.09	3.50	< .001	1.35	1.14	1.60
Maternal older sisters	0.07	0.08	0.85	.395	1.07	0.91	1.27
Nonmaternal younger brothers	0.00	0.12	0.02	.986	1.00	0.79	1.27
Nonmaternal younger sisters	− 0.14	0.17	− 0.82	.413	0.87	0.63	1.21
Nonmaternal older brothers	0.07	0.17	0.40	.693	1.07	0.77	1.48
Nonmaternal older sisters	0.34	0.17	1.98	.047	1.41	1.00	1.97
Age	0.00	0.00	− 0.72	.473	1.00	0.99	1.01
Mother’s age at target’s birth	− 0.07	0.01	− 4.82	< .001	0.94	0.91	0.96

Estimates represent the log odds using heterosexuals as a referential value

When we applied the novel parameterization (all VIFs < 5) published by Ablaza et al. (2022), the effect of maternal older brothers decreased in magnitude (*b* = 0.23, *p* = 0.050, OR = 1.26 [95% CI: 1.00, 1.60]) and was now bordering the level of standard formal significance (see Table 5). We have also recorded two significant relationships: larger sibship size decreased the odds of homosexuality (*b* = -0.40, *p* < 0.001, OR = 0.67 [95% CI: 0.55, 0.81]), while having more older siblings (interpreted here as an increase in the number of older sisters with the number of older brothers being fixed, i.e., the SBOE; see Ablaza et al., 2022) had increased the odds of homosexuality (*b* = 0.46, *p* < 0.001, OR = 1.59 [95% CI: 1.26, 2.01]).

**Table 5:** Novel parameterization of the fraternal birth order effect in men

						95% CI	
	*b*	*SE*	*Z*	*p*	OR	Lower	Upper
Intercept	1.73	0.42	4.13	< .001	5.65	2.49	12.86
Sibship size	− 0.40	0.10	− 4.16	< .001	0.67	0.55	0.81
Maternal older siblings	0.46	0.12	3.86	< .001	1.59	1.26	2.01
Maternal older brothers	0.23	0.12	1.96	.050	1.26	1.00	1.60
Maternal younger brothers	0.20	0.12	1.62	.106	1.22	0.96	1.54
Age	− 0.00	0.00	− 0.80	.423	1.00	0.99	1.01
Mother’s age at target’s birth	− 0.06	0.01	− 4.67	< .001	0.94	0.91	0.96

Estimates represent the log odds using heterosexuals as a referential value

We used the Wilcoxon-signed rank test to see whether the excess of older brothers (according to a conventional parameterization) could be attributed to maternal effects. If the maternal immune hypothesis is correct, we would expect to see more maternal than paternal older half-brothers in the sample of gay men and no difference in the sample of straight men. But in our analysis, the difference between the number of maternal older half-brothers (*M* = 0.07, *SD* = 0.29) and paternal older half-brothers (*M* = 0.05, *SD* = 0.25) in the sample of gay men did not reach statistical significance (*p* = 0.134). We did, however, find a significant difference in the number of maternal older half-sisters (*M* = 0.04, *SD* = 0.23) and paternal older half-sisters (*M* = 0.08, *SD* = 0.30) in gay men (*p* = 0.002) (see Table 6).

**Table 6:** Maternal and paternal half-brothers and half-sisters in gay and straight men

Maternal half siblings			Paternal half siblings						
	*M*	*SD*		*M*	*SD*	*N*	*W*	*p*	*d*
Older brothers gay	0.07	0.29	Older brothers gay	0.05	0.25	698	1323	.134	.06
Older brothers straight	0.03	0.19	Older brothers straight	0.06	0.28	843	449	.032	.07
Older sisters gay	0.04	0.23	Older sisters gay	0.08	0.30	698	535	.002	.11
Older sisters straight	0.03	0.20	Older sisters straight	0.05	0.24	843	504	.070	.07

### Anal-Erotic Role Orientation

For AERO behavior, we found that only age is significantly higher in tops (*M* = 36.65, *SD* = 11.20) than in bottoms (*M* = 33.68, *SD* = 11.45, *t* = 2.24, *p* = 0.026). None of the tested variables were significantly different between tops and bottoms for AERO preferences.

We used a conventionally parameterized binary logistic regression model to test the hypothesized relationship between maternal older brothers and other siblings and AERO behavior and AERO preferences, respectively. For AERO behavior (all VIFs < 2), we found no statistically significant effect of any of the independent variables, including maternal older brothers (see Table 7). Contrary to our predictions, tops (*N* = 133) showed a nonsignificant excess of older brothers compared to bottoms (*N* = 160). The OBOR statistic (with bottoms set as the “exposed” group) yielded a nonsignificant result (*z* = 0.99, *p* = 0.32, OBOR = 0.80 [95% CI: 0.51, 1.24]). Results of the novel parameterization (Ablaza et al., 2022), which are shown in Table 8, delivered no significant result (the highest VIF = 5.02 for maternal older siblings, all other VIFs < 5).

**Table 7:** Anal-erotic role orientation behavior in gay men

						95% CI	
	*b*	*SE*	*Z*	*p*	OR	Lower	Upper
Intercept	0.45	0.94	0.48	.630	1.57	0.25	9.86
Maternal younger brothers	− 0.18	0.18	− 1.02	.306	0.83	0.59	1.18
Maternal younger sisters	0.01	0.24	0.05	.964	1.01	0.63	1.61
Maternal older brothers	− 0.38	0.20	− 1.87	.062	0.68	0.46	1.02
Maternal older sisters	− 0.03	0.19	− 0.18	.857	0.97	0.66	1.40
Nonmaternal younger brothers	− 0.09	0.27	− 0.34	.736	0.91	0.54	1.55
Nonmaternal younger sisters	1.22	0.64	1.90	.057	3.40	0.96	11.96
Nonmaternal older brothers	− 0.16	0.40	− 0.41	.684	0.85	0.39	1.87
Nonmaternal older sisters	0.45	0.45	1.01	.311	1.58	0.65	3.80
Age	− 0.02	0.01	− 1.99	.047	0.98	0.96	1.00
Mother’s age at target’s birth	0.02	0.03	0.79	.432	1.02	0.96	1.09

Estimates represent the log odds using tops as a referential value

**Table 8:** Novel parameterization of anal-erotic role orientation behavior in gay men

						95% CI	
	*b*	*SE*	*Z*	*p*	OR	Lower	Upper
Intercept	0.59	0.90	0.65	.515	1.80	0.31	10.59
Sibship size	0.06	0.23	0.24	.811	1.06	0.67	1.66
Maternal older siblings	− 0.12	0.28	− 0.44	.656	0.88	0.51	1.52
Maternal older brothers	− 0.29	0.27	− 1.07	.285	0.75	0.44	1.27
Maternal younger brothers	− 0.28	0.27	− 1.02	.307	0.76	0.45	1.29
Age	− 0.02	0.01	− 2.22	.026	0.98	0.96	1.00
Mother’s age at target’s birth	0.02	0.03	0.82	.411	1.03	0.97	1.09

Estimates represent the log odds using tops as a referential value

Table 9 shows the results of a conventionally parameterized regression for AERO preferences (all VIFs < 2). Tops (*N* = 120) showed a nonsignificant excess of older brothers compared to bottoms (*N* = 200). The OBOR statistic yielded a nonsignificant result (*z* = 0.03, *p* = 0.98, OBOR = 1.01 [95% CI: 0.67, 1.50]). The model that used the novel parameterization yielded no significant result (see Table 10; the highest VIF = 6.13 for maternal older siblings, all other VIFs < 5).

**Table 9:** Anal-erotic role orientation preferences in gay men

						95% CI	
	*b*	*SE*	*Z*	*p*	OR	Lower	Upper
Intercept	0.71	0.96	0.73	.463	2.03	0.31	13.43
Maternal younger brothers	− 0.21	0.21	− 1.02	.306	0.81	0.54	1.21
Maternal younger sisters	− 0.21	0.25	− 0.86	.391	0.81	0.49	1.32
Maternal older brothers	− 0.27	0.17	− 1.60	.110	0.76	0.55	1.06
Maternal older sisters	− 0.38	0.19	− 2.02	.043	0.68	0.47	0.99
Nonmaternal younger brothers	− 0.09	0.30	− 0.28	.776	0.92	0.51	1.66
Nonmaternal younger sisters	0.49	0.45	1.09	.276	1.63	0.68	3.93
Nonmaternal older brothers	− 0.22	0.35	− 0.63	.527	0.80	0.40	1.59
Nonmaternal older sisters	0.38	0.39	0.99	.323	1.46	0.69	3.11
Age	− 0.01	0.01	− 1.17	.242	0.99	0.97	1.01
Mother’s age at target’s birth	0.02	0.03	0.64	.520	1.02	0.96	1.09

Estimates represent the log odds using tops as a referential value

**Table 10:** Novel parameterization of anal-erotic role orientation preferences in gay men

						95% CI	
	*b*	*SE*	*Z*	*p*	OR	Lower	Upper
Intercept	0.76	0.93	0.81	.418	2.13	0.34	13.27
Sibship size	− 0.21	0.25	− 0.85	.398	0.81	0.50	1.32
Maternal older siblings	− 0.17	0.28	− 0.62	.536	0.84	0.48	1.46
Maternal older brothers	0.12	0.24	0.49	.623	1.12	0.71	1.79
Maternal younger brothers	− 0.01	0.30	− 0.03	.978	0.99	0.55	1.79
Age	− 0.01	0.01	− 1.25	.213	0.99	0.97	1.01
Mother’s age at target’s birth	0.02	0.03	0.68	.498	1.02	0.96	1.08

Estimates represent the log odds using tops as a referential value

In straight men with bottom AERO, we tested for a potential presence of the FBOE in the same way as in gay men. None of the sociodemographic variables were significantly different between tops and bottoms for either AERO behavior or AERO preferences. Using the conventionally parameterized model, we found no statistically significant effect of any class of siblings on AERO (AERO behavior *N*_tops_ = 211, *N*_bottoms_ = 33; AERO preferences *N*_tops_ = 345, *N*_bottoms_ = 104; all VIFs < 2 for both conventionally parameterized models). The OBOR statistic yielded nonsignificant results both for straight AERO behavior (*z* = 1.65, *p* = 0.10, OBOR = 0.44 [95% CI: 0.17, 1.17]) and straight AERO preferences (*z* = 0.14, *p* = 0.89, OBOR = 0.97 [95% CI: 0.62, 1.52]).

Using the novel parameterization, we found no significant result for AERO behavior in straight men (all VIFs < 4). Interestingly, though, it revealed for AERO preferences in straight men (all VIFs < 4) a significant effect of sibship size (*b* = 0.43, *p* = 0.010, OR = 1.53 [95% CI: 1.10, 2.12]), implying that larger sibship size increases straight man’s odds of being labeled as a bottom. See Supplementary Tables S14–S17 for complete results regarding straight men.

### The Fraternal Birth Order Effect in Women

Descriptive statistics of the female sample are summarized in Tables 11 and 12. We found significant differences in participants’ age (*t* = 5.94, *p* < 0.001), the importance of religious faith (*t* = 5.68, *p* < 0.001), and in the population size of the place of residence in childhood (χ^2^ = 10.76, *p* = 0.029), see Tables 11 and 12 for details. Descriptive statistics on lesbian and straight women’s siblings are presented in Table 13.

**Table 11:** Descriptive statistics for age and religiousness in the female sample

	Sexual orientation	*N*	Missing	*M*	*SD*	Median	*p*
Age	Straight	331	0	31.77	9.78	30.00	< .001
	Lesbian	265	0	27.75	6.71	26.00	
Importance of religious faith (0–100)^a^	Straight	302	29	26.93	30.87	12.50	< .001
	Lesbian	227	38	13.59	23.13	0.00	

^a^Where 0 means “not important” and 100 “very important”

**Table 12:** Descriptive statistics for education and population sizes of places of residence in the female sample

	Level	Sexual orientation				Total	*p*
		Straight		Lesbian			
		*N*	%	*N*	%		
Education	Primary	5	1.5	11	4.2	16	< .001
	Upper secondary (vocational)	7	2.1	22	8.3	29	
	Upper secondary (high school graduation)	122	36.9	140	52.8	262	
	Tertiary (bachelor’s degree or equivalent)	77	23.3	50	18.9	127	
	Tertiary (master’s degree or equivalent)	102	30.8	38	14.3	140	
	Tertiary (doctorate or equivalent)	17	5.1	3	1.1	20	
	< missing >	1	0.3	1	0.4	2	
Population size of place of residence in childhood	< 1000	39	11.8	45	17.0	84	.029
	1000–5000	53	16.0	39	14.7	92	
	5000–50,000	96	29.0	88	33.2	184	
	50,000–500,000	73	22.1	59	22.3	132	
	> 500,000	68	20.5	31	11.7	99	
	< missing >	2	0.6	3	1.1	5	
Population size of place of residence (current)	< 1000	30	9.1	18	6.8	48	.061
	1000–5000	24	7.3	27	10.2	51	
	5000–50,000	61	18.4	57	21.5	118	
	50,000–500,000	77	23.3	78	29.4	155	
	> 500,000	127	38.4	77	29.1	204	
	< missing >	12	3.6	8	3.0	20	
Total			331		265	596

**Table 13:** The number of siblings of straight and lesbian women

	Sexual orientation					
	Straight			Lesbian		
	*N*^a^	*M*	*SD*	*N*^a^	*M*	*SD*
Maternal younger brothers	331	0.35	0.64	265	0.35	0.68
Maternal older brothers	331	0.28	0.53	265	0.37	0.66
Maternal younger sisters	331	0.32	0.58	265	0.35	0.58
Maternal older sisters	331	0.30	0.54	265	0.32	0.68
Nonmaternal younger brothers	331	0.13	0.40	265	0.22	0.57
Nonmaternal older brothers	331	0.11	0.39	265	0.18	0.53
Nonmaternal younger sisters	331	0.13	0.42	265	0.11	0.40
Nonmaternal older sisters	331	0.11	0.41	265	0.17	0.53

^a^There were no missing values in any of the variables

A conventionally parameterized binary logistic regression (all VIFs < 2) revealed that the number of maternal older brothers has a significant positive effect on female homosexual orientation (*b* = 0.54, *p* = 0.001, OR = 1.71 [95% CI: 1.23, 2.37]), see Table 14. The OBOR statistic for women, however, yielded a nonsignificant result (*z* = 1.27, *p* = 0.20, OBOR = 1.23 [95% CI: 0.89, 1.71]). After reanalyzing the data with the novel parameterization (all VIFs < 5), the estimate of maternal older brothers decreased and ceased to be significant (*b* = 0.32, *p* = 0.120, OR = 1.38 [95% CI: 0.92, 2.07]; see Table 15.

**Table 14:** Fraternal birth order effect in women

						95% CI	
	*b*	*SE*	*Z*	*p*	OR	Lower	Upper
Intercept	3.84	0.73	5.24	< .001	46.72	11.10	196.70
Maternal younger brothers	− 0.08	0.14	− 0.62	.538	0.92	0.70	1.20
Maternal younger sisters	0.03	0.16	0.17	.863	1.03	0.75	1.40
Maternal older brothers	0.54	0.17	3.23	.001	1.71	1.23	2.37
Maternal older sisters	0.25	0.16	1.58	.114	1.29	0.94	1.76
Nonmaternal younger brothers	0.30	0.19	1.55	.120	1.35	0.92	1.97
Nonmaternal younger sisters	− 0.22	0.23	− 0.99	.322	0.80	0.51	1.24
Nonmaternal older brothers	0.30	0.21	1.44	.149	1.35	0.90	2.03
Nonmaternal older sisters	0.26	0.20	1.27	.204	1.30	0.87	1.93
Age	− 0.06	0.01	− 5.61	< .001	0.94	0.92	0.96
Mother’s age at target’s birth	− 0.09	0.02	− 4.18	< .001	0.91	0.87	0.95

Estimates represent the log odds using heterosexuals as a referential value

**Table 15:** Novel parameterization of fraternal birth order effect in women

						95% CI	
	*b*	*SE*	*Z*	*p*	OR	Lower	Upper
Intercept	3.93	0.72	5.47	< .001	51.16	12.48	209.65
Sibship size	0.01	0.16	0.09	.927	1.01	0.75	1.38
Maternal older siblings	0.23	0.22	1.07	.286	1.26	0.82	1.94
Maternal older brothers	0.32	0.21	1.56	.120	1.38	0.92	2.07
Maternal younger brothers	− 0.08	0.20	− 0.41	.684	0.92	0.63	1.36
Age	− 0.07	0.01	− 5.80	< .001	0.94	0.92	0.96
Mother’s age at target’s birth	− 0.09	0.02	− 4.16	< .001	0.91	0.87	0.95

Estimates represent the log odds using heterosexuals as a referential value

### Follow-Up Analyses

An anonymous reviewer drew our attention to an important issue discussed also in Blanchard (2022). In the Ablaza et al.'s, (2022, Table 1) parameterization, the focal parameter of interest, identified as the FBOE, is the number of older brothers which “…corresponds to a situation in which one older sister is replaced by an older brother in an existing sibship.” But replacement of an older sister by an older brother does not necessarily result in the genuine FBOE: it rather yields something what Blanchard (2022) calls the fraternal-sororal effect differential (FSED). And, if there is some evidence suggesting that older sisters could also have some effect on sexual orientation (Blanchard et al., 2021), replacement of an older sister for an older brother could be rather compared to replacing a parameter with a weaker effect by a parameter that has a stronger effect; it does not amount to replacing a parameter with no effect by a parameter that has some effect.

Bearing this in mind, we have re-run the two main models (full male sample and full female sample) in a modified form where we replaced the predictors corresponding to the numbers of younger brothers and older brothers by predictors corresponding to younger sisters and older sisters. In this parameterization, the predictor of older siblings would correspond to a situation where an individual has one older brother more and one younger brother less, which should thus reflect the FBOE as typically understood in the literature (see also Table 1 in Blanchard, 2022). This parameterization has already been used to test the influence of the FBOE on handedness (Bartlett et al., 2024). The results of this “revised novel parameterization” are shown in Tables 16, 17 for the male and female sample, respectively.

**Table 16:** Revised novel parameterization of the fraternal birth order effect in men

						95% CI	
	*b*	*SE*	*Z*	*p*	OR	Lower	Upper
Intercept	1.73	0.42	4.13	< .001	5.65	2.49	12.86
Sibship size	− 0.21	0.08	− 2.53	.011	0.81	0.69	0.95
Maternal older siblings	0.50	0.11	4.47	< .001	1.65	1.33	2.06
Maternal older sisters	− 0.23	0.12	− 1.96	.050	0.79	0.63	1.00
Maternal younger sisters	− 0.20	0.12	− 1.62	.106	0.82	0.65	1.04
Age	− 0.00	0.00	− 0.80	.423	1.00	0.99	1.01
Mother’s age at target’s birth	− 0.06	0.01	− 4.67	< .001	0.94	0.91	0.96

Estimates represent the log odds using heterosexuals as a referential value

**Table 17:** Revised novel parameterization of fraternal birth order effect in women

						95% CI	
	*b*	*SE*	*Z*	*p*	OR	Lower	Upper
Intercept	3.93	0.72	5.47	< .001	51.16	12.48	209.65
Sibship size	− 0.07	0.14	− 0.49	.626	0.94	0.72	1.22
Maternal older siblings	0.63	0.21	3.05	.002	1.89	1.25	2.84
Maternal older sisters	− 0.32	0.21	− 1.56	.120	0.73	0.48	1.09
Maternal younger sisters	0.08	0.20	0.41	.684	1.08	0.74	1.60
Age	− 0.07	0.01	− 5.80	< .001	0.94	0.92	0.96
Mother’s age at target’s birth	− 0.09	0.02	− 4.16	< .001	0.91	0.87	0.95

Estimates represent the log odds using heterosexuals as a referential value

For men, the revised novel parameterization (all VIFs < 5) revealed a significant effect of the number of maternal older siblings, i.e., the FBOE (*b* = 0.50, *p* < 0.001, OR = 1.65 [95% CI: 1.33, 2.06]). For women, the revised novel parameterization (all VIFs < 4) had likewise shown an effect of maternal older siblings (*b* = 0.63, *p* = 0.002, OR = 1.89 [95% CI: 1.25, 2.84]).

## Discussion

The main aim of this paper was to replicate the presence of the FBOE among gay men, replicate the recent finding regarding the presence of the FBOE in lesbian women, inspect whether the FBOE could be specific only to sexually receptive gay men, and explore whether the FBOE might be present also among sexually receptive straight men. We used multiple differently parameterized logistic regression models to test each of our assumptions. Our positive findings regarding the FBOE in both homosexual men and women (as opposed to their straight counterparts) are, nonetheless, not without ambiguity: on the one hand, both the conventional and the more nuanced revised novel parameterization (Blanchard, 2022) brought the expected results and demonstrated the FBOE for both gay men and lesbian women. On the other hand, the novel parameterization proposed by Ablaza et al. (2022) yielded a null result for women and a trend in the expected direction for men. We found no evidence of the FBOE in either gay or straight sexually receptive men. Our research is important since it is the first one to test the relationship of the FBOE and sexual orientation in a Slavic (Czech and Slovak) sample and one of but a handful of studies to explore the potential presence of the FBOE in a female sample.

### The Fraternal Birth Order Effect in Men

An interesting result emerged from a comparison of the three different parameterizations used to model the relevant relationships. Results of the conventional parameterization, commonly employed by other researchers in the field, support the previously established finding of the FBOE in gay men, i.e., they indicate that gay men have more older brothers than straight men do. The novel parameterization, however, decreased the estimate and the effect of older brothers (the relevant predictor) on male sexual orientation then ceased to be significant. It should be noted, though, that the magnitude of the effect (the odds ratio) did not decrease by much and was still greater than one (it went from 1.35 for the conventional parameterization to 1.26 for the novel parameterization). This is important because in Ablaza et al.’s article (2022) the estimate delivered by the novel parameterization was significantly larger than the estimate delivered by the conventional parameterization. Ablaza et al., (2022, p. 680) suggested that the previous null results concerning the FBOE may be overturned by analyses based on their novel parameterization. Our results hint at the opposite, although it should be noted that the low statistical precision of our estimates prevents us from rigorously assessing the relative magnitude of the novel and conventional estimates, which in our study (in contrast to Ablaza et al., 2022) did not significantly differ from each other. It thus seems at least possible that application of the novel parameterization to other previous FBOE studies might nullify some of their positive results as well. But that would need to be tested, possibly through a meta-analysis of all previously published studies on the FBOE that would apply the novel parameterization. Such endeavor would be more than welcome.

One could object that the four predictors of nonmaternal siblings (nonmaternal older and younger brothers and sisters) that were included in the conventionally parameterized model are missing in the novel parameterization, which may have led to incomparable results. That is why we re-ran all the novel models including these four predictors, but the results did not change in any important respect and remained approximately the same.

Although the novel parameterization did not support the FBOE, the revised novel parameterization (Blanchard, 2022) did. This parametrization was applied in response to the insightful feedback received during the review process. In our view, it may be even better suited to differentiate between a possible presence of the FBOE and other intervening effects. Nevertheless, it is rather difficult to draw any unambiguous conclusion from these results. If anything, our results indicate that the FBOE can be harder to detect in some specific samples or populations and by certain specific analyses. We want to stress, though, that using both the conventional parameterization and the revised version of the novel parameterization we have demonstrated the effect reported by numerous previous studies. Additionally, we emphasize that the novel parameterization (Ablaza et al., 2022) and the revised novel parameterization (Blanchard, 2022) are not two different statistical approaches but rather the same approach used in two different versions to estimate effects that cannot be estimated with a single version. The parameters identified as the FBOE are different in the novel and the revised novel parameterization–in the former case, the FBOE is identified as substituting an older brother for an older sister, whereas in the latter case, the FBOE is identified as substituting an older brother for a younger brother (see above).

In accordance with some other studies (Ablaza et al., 2022; Blanchard et al., 2021), we found a significant association between sexual orientation and the predictor of older siblings in the novel parameterization (this actually corresponds to the number of older sisters while the number of older brothers is fixed or the SBOE, see the Results and Ablaza et al., 2022).^1^

Another significant predictor in the novel parameterization is the total sibship size, which shows that gay men have smaller sibships than straight men do. This runs counter the results of some studies which reported that mothers of gay men are more fertile than mothers of straight men (Camperio Ciani & Pellizzari, 2012; Camperio Ciani et al., 2004; Iemmola & Camperio Ciani, 2009). Those initial findings were used to develop the so-called sexually antagonistic gene hypothesis, which provided an evolutionary explanation for the persistence of male homosexuality in human population (for details, see Camperio Ciani et al., 2015). Nevertheless, more recent studies (including the present one) have not replicated those results and the hypothesis has been lately challenged on both empirical and theoretical grounds (Ablaza et al., 2022; Blanchard et al., 2020; Raymond et al., 2023; Semenyna et al., 2023; but see also Song & Zhang, 2023 or Zietsch et al., 2021).

In the conventionally parameterized model, our results showed a negative association between the number of maternal younger sisters and homosexuality. Other studies, too, indeed sometimes show this negative association between the number of younger siblings and male homosexuality (Blanchard & Lippa, 2021). One possible explanation for this effect is that the odds of homosexuality may also be increased in only-child men (Blanchard & Lippa, 2021) who, by definition, have no siblings. But it is also possible that this negative association is due to some artificial bias concerning the female-favoring stopping rule specific to our sample, because we found the proportion of males among gay men’s younger siblings to be significantly higher than expected (see Table S13 and Follow-Up Analyses Regarding Siblings’ Sex Proportion in Supplementary material).

Gay men in our sample did not show an excess of nonmaternal older brothers. An excess of nonmaternal older brothers in gay men might indicate that older brothers influence their younger brothers’ sexuality in a social, nonbiological way because they cannot provoke an immunological response in the individual’s mother and thus alter the prenatal environment. We should note, however, that in our study, nonmaternal siblings were calculated as a sum of paternal half-siblings and stepsiblings “with whom the participants have been raised during at least part of their childhood.” Our classification of nonmaternal siblings is therefore by no means as good as it was in the study of Bogaert (2006), who had exact data on how long participants were raised together in childhood. Bogaert (2006) also intentionally sampled participants reared in nonbiological families, whereas we excluded adoptees because their low number would render any additional analysis impossible. For these reasons, we are reluctant to discard the hypothesis of nonmaternal older brothers increasing the odds of homosexuality in men.

Additionally, it should be borne in mind that fertility rates in WEIRD populations have been consistently decreasing over the past decades. The widespread use of contraceptives and family planning methods, both of which contribute to reduced fertility, may quite possibly mask some effects observed in ancestral environments (Song & Zhang, 2023), including the presumed FBOE. This is because a smaller family size would also result in a lower number of male children born to a mother, thereby reducing her exposure to Y-linked antigens. Despite this limitation, the FBOE has been previously recorded in both WEIRD (e.g., Ablaza et al., 2022; Apostolou, 2020; Blanchard & Bogaert, 1996) and non-WEIRD samples (e.g., Gómez Jiménez et al., 2020; VanderLaan & Vasey, 2011).

### Anal-Erotic Role Orientation

Regarding the FBOE and AERO in gay men, we found no significant results in any of our regression models. This applies to both AERO preferences and AERO behavior, using both conventional and novel parameterization. Surprisingly, when examining the conventionally parameterized AERO behavior, we observed that tops tended to have non-significantly more maternal older brothers than bottoms. This finding partially aligns with the results of Swift-Gallant et al. (2018), who likewise found a nonsignificant excess of older brothers among tops for AERO behavior as compared to bottoms. They also discovered a higher number of older brothers among bottoms for AERO preferences, compared to the general population, but found no significant results for gay men who engage in bottom *behavior* compared to the general population (Swift-Gallant et al., 2018). On the other hand, our results differ from those published by Wampold (2018b), who found among gay men who engage in bottom behavior an excess of older brothers compared to those who engage in top behavior. In line with our findings, though, Wampold (2018b) found no significant results for preferential bottoms.

Our results also somewhat undermine the concept of different etiological subgroups of gay men (Swift-Gallant et al., 2019). Supposedly, gays expressing the FBOE form a distinct group among gay men and display some feminine traits (Swift-Gallant et al., 2021; Wampold, 2013). While we did not measure gender nonconformity or the finger length ratios of our participants and cannot therefore tell whether they had these feminine traits, based on AERO we found no distinct group of gay men to whom the FBOE applies. This could mean that our results are falsely negative, that the concept of different etiological origins of male homosexuality is incorrect, or that the notion of different etiological origins of male homosexuality is correct but dividing gay men according to their AERO is not a good approach to defining possible subgroups. We are unable to tell which of the explanations above is correct and must therefore surmise that the results published by Swift-Gallant et al. (2018) and by Wampold (2018b) are, in conjunction with our findings, inconclusive.

Regarding the FBOE and AERO in straight men, once again we obtained no significant results for AERO behavior, neither with the conventional or with the novel parameterization. For AERO preferences, we obtained no significant results in the conventionally parameterized model, but one significant result emerged when we used the novel parameterization for AERO preferences: we found that bottom straight men tend to have larger sibship sizes than top straight men. We are not sure how to interpret this finding but note the younger sibling sex ratio was severely distorted in straight men with bottom AERO preferences (see Table S13), with a great predominance of younger sisters over younger brothers.

All in all, these results somewhat contradict the reasoning of Wampold (2018a), according to whom the FBOE might be associated not with male homosexuality as such but rather with the wish to be anally penetrated. Our results regarding straight men are, however, of limited importance due to the small sample size (e.g., only 33 bottom straight men in AERO behavior) and the necessity to include versatiles, i.e., individuals who indicated an about an equal preference for bottom and top behavior, into the bottom group to increase its size. While in gay AERO behavior, we only included gay men who indicated they had anal intercourse at least 30 times (thus setting a relatively stringent threshold for experience with anal sex), for straight AERO behavior we lowered this threshold to having experienced anal intercourse at least three times. Due to these limitations, our data on straight men’s AERO should be interpreted with caution. 

### The Fraternal Birth Order Effect in Women

Our findings for the FBOE in women are somewhat similar to the situation in the male sample: both the conventional parameterization and the revised novel parameterization (Blanchard, 2022) showed the significant FBOE but the novel parameterization (Ablaza et al., 2022) delivered a null result.

Since there are only a handful of studies dealing with the FBOE in women (see, e.g., Blanchard & Skorska, 2022 or Xu & Zheng, 2017 for a negative result, and Ablaza et al., 2022 for a positive result), it would be premature to draw conclusions about the existence of the FBOE in female population. Still, we hope that our results, together with those of other research teams, will encourage others to explore the topic further. If replicated, the presence of the FBOE in women would pose a challenge for the maternal immune hypothesis, which was originally proposed as a sex-asymmetrical immunological explanation of male homosexuality (see Introduction). At this point, we refrain from conjecturing about the meaning of the FBOE in lesbian women. Readers who wish to explore the subject further will find additional information in Blanchard and Skorska (2022) and Semenyna et al. (2022).

### Conclusion

We recorded the FBOE for gay men and lesbian women in an online study of Czech and Slovak participants. Our study aligns with a growing body of research that has demonstrated the FBOE in male samples and is one of a few studies that inspected and demonstrated the presence of the FBOE in a female sample. Yet, a slight ambiguity persists due to the non-affirmative result from one of the three different parameterizations we used to estimate the effect, although the odds ratios provided by said parameterization still indicated higher numbers of older brothers in both gay men and lesbian women. The presence of the FBOE in lesbian women has only recently become a topic of serious scientific interest and lesbian women should be routinely included in future studies on the FBOE. Contrary to prior reports, though, we found no evidence for an excess of older brothers in either gay or straight men with receptive AERO; this warrants further scrutiny. Additionally, our findings revealed a difference in the number of offspring born to mothers of gay men compared to those of straight men, offering insights into potential familial patterns related to sexual orientation. For women, though, we found no significant difference in maternal fertility between straight and lesbian women.

We suggest that further studies employing the novel regression parameterization(s) should be conducted on samples of women and men of various sexual orientations, and on samples of men differing with respect to their AERO, to unravel the subtleties of familial influences on sexual orientation. Understanding these associations would contribute to broader insights into the development of human sexual orientation.

### Supplementary Information

Below is the link to the electronic supplementary material.

Supplementary file1 (DOCX 118 kb)

## Data Availability

Data associated with this research and the preregistration are available publicly at Open Science Framework: https://osf.io/ugx6t/.

## Footnotes

1 It should be noted that substituting an older brother for an older sister and substituting an older brother for a younger brother are functionally equivalent procedures if both older sisters and younger brothers have zero effect on the odds of homosexuality. In that case, either operation can be interpreted as estimating the FBOE. If, however, older sisters increase the odds of homosexuality (as in our case, see Table 5) and thus there does exist a sororal birth order effect (SBOE), then substituting an older brother for an older sister does not estimate the magnitude of the FBOE but rather the difference in magnitude between the FBOE and the SBOE, the so-called fraternal-sororal effect differential (FSED; see also Follow-Up Analyses in Results). This claim is further supported by formal simulations of Raymond et al. (2024) indicating that when the SBOE was present in a sample, then “a significant drop in the power” of Ablaza et al.’s (2022) parameterization occurred (in comparison with a situation when no SBOE was present; Raymond et al., 2024, and their Appendix 3). Given this reasoning and the SBOE documented in our sample, the absence of the formally significant FBOE in the novel parameterization need not be a cause for alert–again, we found evidence for the FBOE in men in both the conventional and the revised novel parameterization. We could, however, in a similar vein, interpret the FBOE in men as identified by the revised novel parameterization (Table 16). As noted earlier, in the revised novel parameterization, the predictor of maternal older siblings corresponds to a situation in which an individual has one younger brother less and one older brother more. In the conventional parameterization (Table 4), we have recorded a significance-approaching trend of gay men having fewer younger brothers than straight men. In this case, substituting one younger brother for an older brother could also be compared to replacing a parameter with a negative effect by a parameter with a positive effect. If our assumption is right, we suppose this may have contributed to the effect size of the FBOE in the revised novel parameterization, which was the largest of all three parameterizations and significantly larger than the FBOE as identified by the novel parameterization (Table 5; based on 95% CIs comparison).
