# Supplementary material for: Examining the Fraternal Birth Order Effect and Sexual Orientation: Insights from an East European Population
Source: Arch Sex Behav. 2024 Jun 13;53(8):2905–22. doi: 10.1007/s10508-024-02892-8 (PMC11335834; doi:10.1007/s10508-024-02892-8)
Supplement: Supplementary file 1 — Supplementary file1 (DOCX 118 kb) [file 10508_2024_2892_MOESM1_ESM.docx]

**SUPPLEMENTARY MATERIAL**

**Supplementary Methods**

To ensure a higher data validity and to control for potential outliers, we have excluded from further analyses participants who reported: having more than nine children, having more than eight full siblings, and/or their mother or father having more than nine children including the participant. Moreover, we have replaced blank cells in the variable “importance of religious faith” with arithmetic means separately for each group (homosexual or heterosexual men or women). Blank cells in variables size of place of residence in childhood and at present were replaced with arithmetic means rounded to the nearest integer.

**Follow-Up Analyses Regarding Siblings’ Sex Proportion**

As Blanchard and Skorska (2022) have described, stopping rules are artificial criteria related to reproduction, which are used by parents if they, for example, want to have at least one child of a certain sex (male-favoring or female-favoring stopping rule), have at least one child of each sex, etc. We analyzed the proportions of brothers for younger and older siblings separately for both groups compared in each of the regression models. A typical naturally occurring sex ratio (i.e., when no stopping rule is employed) is 106 males per 100 females. This can also be expressed as a proportion of brothers, i.e., 106 brothers per 206 total siblings, which corresponds to 0.515. To test whether the proportions of male siblings in our sample significantly departs from this theoretical threshold, we used a statistical test for one proportion, which is available online here: <https://www.medcalc.org/calc/test_one_proportion.php>. The complete results are summarized in Table S13. The proportion of brothers among straight men’s older siblings was 0.494, which did not significantly depart from the expected value of 0.515 (*p* = .345). The same holds for the proportion of brothers among straight men’s younger siblings, which was 0.532 (*p* = .385). The proportion of brothers among gay men’s older siblings was 0.546 (*p* = .154), while the proportion of brothers among gay men’s younger siblings was 0.572 (*p* = .017). Three other cases significantly departed from the expected value of 0.515, namely: the proportion of older siblings of bottom straight men in AERO behavior analysis (proportion of brothers 0.25, *p* = .018), the proportion of younger siblings of top gay men in AERO behavior analysis (proportion of brothers 0.624, *p* = .036), and the proportion of younger siblings of bottom straight men in AERO preferences analysis (proportion of brothers 0.409, *p* < .001).

Figure S1: Kinsey scale and four related items

| 1 | “How would you define yourself on the scale shown below?” |
| --- | --- |
|  | *(Scale 0–6; 0 – certainly heterosexual, 6 – certainly homosexual)* |
| 2 | “If you desired sexual intercourse at a party, you would choose:” |
|  | *(Scale 0–6; 0 – always and only the opposite sex, 6 – always and only the same sex)* |
| 3 | “When you fantasize sexual intercourse, your sexual partners are:” |
|  | *(Scale 0–6; 0 – always and only of the opposite sex, 6 – always and only of the same sex)* |
| 4 | “Your sexual partners during the past year were:” |
|  | *(Scale 0–6; 0 – always and only of the opposite sex, 6 – always and only of the same sex)* |
| 5 | “Your sexual partners during the last five years were:” |
|  | *(Scale 0–6; 0 – always and only of the opposite sex, 6 – always and only of the same sex)* |

Table S1: Fraternal birth order effect in men (covariates as preregistered)

|  | | | | | | 95% CI | |
| --- | --- | --- | --- | --- | --- | --- | --- |
|  | *b* | *SE* | *Z* | *p* | OR | Lower | Upper |
| Intercept | 0.47 | 0.21 | 2.23 | .025 | 1.60 | 1.06 | 2.41 |
| Maternal younger brothers | -0.10 | 0.08 | -1.25 | .210 | 0.90 | 0.77 | 1.06 |
| Maternal younger sisters | -0.31 | 0.10 | -3.15 | .002 | 0.73 | 0.61 | 0.89 |
| Maternal older brothers | 0.25 | 0.09 | 2.87 | .004 | 1.28 | 1.08 | 1.51 |
| Maternal older sisters | 0.02 | 0.08 | 0.20 | .842 | 1.02 | 0.86 | 1.20 |
| Nonmaternal younger brothers | 0.10 | 0.13 | 0.78 | .436 | 1.10 | 0.86 | 1.41 |
| Nonmaternal younger sisters | -0.09 | 0.17 | -0.53 | .594 | 0.91 | 0.66 | 1.27 |
| Nonmaternal older brothers | 0.00 | 0.17 | 0.00 | .997 | 1.00 | 0.72 | 1.39 |
| Nonmaternal older sisters | 0.36 | 0.17 | 2.08 | .037 | 1.43 | 1.02 | 2.01 |
| Population size of place of residence (childhood)^a^  2–1 | -0.51 | 0.22 | -2.31 | .021 | 0.60 | 0.39 | 0.92 |
| 3–1 | -0.45 | 0.21 | -2.20 | .028 | 0.64 | 0.43 | 0.95 |
| 4–1 | -0.91 | 0.21 | -4.33 | < .001 | 0.40 | 0.27 | 0.61 |
| 5–1 | -1.04 | 0.24 | -4.31 | < .001 | 0.35 | 0.22 | 0.57 |
| Population size of place of residence (present)^a^  2–1 | -0.09 | 0.27 | -0.34 | .734 | 0.91 | 0.54 | 1.54 |
| 3–1 | -0.09 | 0.25 | -0.35 | .726 | 0.92 | 0.56 | 1.50 |
| 4–1 | 0.22 | 0.23 | 0.98 | .328 | 1.25 | 0.80 | 1.95 |
| 5–1 | 0.63 | 0.23 | 2.68 | .007 | 1.87 | 1.18 | 2.95 |
| Importance of religious faith | -0.01 | 0.00 | -5.71 | < .001 | 0.99 | 0.99 | 0.99 |

Note: Estimates represent the log odds using heterosexuals as a referential value.

^a^ Rows represent a comparison against a baseline (“1”), which is the option with the lowest number of inhabitants. See description of relevant variables in Methods.

Table S2: Novel parameterization of the fraternal birth order effect in men (covariates as preregistered)

|  |  |  |  |  |  | 95% CI | |
| --- | --- | --- | --- | --- | --- | --- | --- |
|  | *b* | *SE* | *Z* | *p* | OR | Lower | Upper |
| Intercept | 0.49 | 0.21 | 2.36 | .018 | 1.64 | 1.09 | 2.47 |
| Sibship size | -0.32 | 0.10 | -3.28 | .001 | 0.73 | 0.60 | 0.88 |
| Maternal older siblings | 0.33 | 0.12 | 2.78 | .005 | 1.39 | 1.10 | 1.74 |
| Maternal older brothers | 0.23 | 0.12 | 1.93 | .054 | 1.26 | 1.00 | 1.61 |
| Maternal younger brothers | 0.21 | 0.12 | 1.70 | .089 | 1.23 | 0.97 | 1.57 |
| Population size of place of residence (childhood)^a^ |  |  |  |  |  |  |  |
| 2–1 | -0.51 | 0.22 | -2.31 | .021 | 0.60 | 0.39 | 0.92 |
| 3–1 | -0.44 | 0.21 | -2.16 | .031 | 0.64 | 0.43 | 0.96 |
| 4–1 | -0.88 | 0.21 | -4.20 | < .001 | 0.41 | 0.28 | 0.63 |
| 5–1 | -1.02 | 0.24 | -4.24 | < .001 | 0.36 | 0.22 | 0.58 |
| Population size of place of residence (present)^a^ |  |  |  |  |  |  |  |
| 2–1 | -0.10 | 0.27 | -0.37 | .711 | 0.91 | 0.54 | 1.53 |
| 3–1 | -0.08 | 0.25 | -0.31 | .760 | 0.93 | 0.57 | 1.52 |
| 4–1 | 0.22 | 0.23 | 0.97 | .332 | 1.25 | 0.80 | 1.95 |
| 5–1 | 0.63 | 0.23 | 2.70 | .007 | 1.88 | 1.19 | 2.97 |
| Importance of religious faith | -0.01 | 0.00 | -5.65 | < .001 | 0.99 | 0.99 | 0.99 |

Note: Estimates represent the log odds using heterosexuals as referential value.

^a^ Rows represent a comparison against a baseline (“1”), which is the option with the lowest number of inhabitants. See description of relevant variables in Methods.

Table S3: AERO behavior in gay men (covariates as preregistered)

|  | | | | | | 95% CI | |
| --- | --- | --- | --- | --- | --- | --- | --- |
|  | *b* | *SE* | *Z* | *p* | OR | Lower | Upper |
| Intercept | 1.10 | 0.44 | 2.49 | .013 | 3.01 | 1.26 | 7.18 |
| Maternal younger brothers | -0.21 | 0.17 | -1.20 | .229 | 0.81 | 0.58 | 1.14 |
| Maternal younger sisters | -0.02 | 0.23 | -0.09 | .931 | 0.98 | 0.62 | 1.55 |
| Maternal older brothers | -0.34 | 0.20 | -1.74 | .082 | 0.71 | 0.48 | 1.04 |
| Maternal older sisters | 0.00 | 0.19 | -0.01 | .995 | 1.00 | 0.69 | 1.44 |
| Nonmaternal younger brothers | -0.13 | 0.27 | -0.48 | .635 | 0.88 | 0.52 | 1.48 |
| Nonmaternal younger sisters | 1.21 | 0.64 | 1.90 | .058 | 3.34 | 0.96 | 11.64 |
| Nonmaternal older brothers | -0.15 | 0.40 | -0.37 | .713 | 0.86 | 0.39 | 1.90 |
| Nonmaternal older sisters | 0.47 | 0.45 | 1.05 | .295 | 1.60 | 0.67 | 3.84 |
| Age | -0.02 | 0.01 | -2.03 | .042 | 0.98 | 0.96 | 1.00 |

Note: Estimates represent the log odds using tops as a referential value.

Table S4: Novel parameterization of AERO behavior in gay men (covariates as preregistered)

|  |  |  |  |  |  | 95% CI | |
| --- | --- | --- | --- | --- | --- | --- | --- |
|  | *b* | *SE* | *Z* | *p* | OR | Lower | Upper |
| Intercept | 1.25 | 0.43 | 2.92 | .003 | 3.47 | 1.51 | 8.01 |
| Sibship size | 0.02 | 0.23 | 0.10 | .922 | 1.02 | 0.65 | 1.60 |
| Maternal older siblings | -0.05 | 0.26 | -0.20 | .838 | 0.95 | 0.57 | 1.59 |
| Maternal older brothers | -0.28 | 0.27 | -1.05 | .292 | 0.75 | 0.45 | 1.27 |
| Maternal younger brothers | -0.27 | 0.27 | -1.00 | .319 | 0.76 | 0.45 | 1.30 |
| Age | -0.02 | 0.01 | -2.27 | .023 | 0.98 | 0.96 | 1.00 |

Note: Estimates represent the log odds using tops as a referential value.

Table S5: AERO preferences in gay men (covariates as preregistered)

|  | | | | | | 95% CI | |
| --- | --- | --- | --- | --- | --- | --- | --- |
|  | *b* | *SE* | *Z* | *p* | OR | Lower | Upper |
| Intercept | 0.86 | 0.21 | 4.12 | < .001 | 2.36 | 1.57 | 3.55 |
| Maternal younger brothers | -0.25 | 0.20 | -1.24 | .216 | 0.78 | 0.53 | 1.15 |
| Maternal younger sisters | -0.26 | 0.24 | -1.05 | .294 | 0.77 | 0.48 | 1.25 |
| Maternal older brothers | -0.24 | 0.16 | -1.50 | .135 | 0.78 | 0.57 | 1.08 |
| Maternal older sisters | -0.36 | 0.18 | -2.01 | .045 | 0.70 | 0.49 | 0.99 |
| Nonmaternal younger brothers | -0.14 | 0.30 | -0.46 | .645 | 0.87 | 0.49 | 1.57 |
| Nonmaternal younger sisters | 0.50 | 0.45 | 1.12 | .262 | 1.65 | 0.69 | 3.97 |
| Nonmaternal older brothers | -0.22 | 0.35 | -0.62 | .535 | 0.80 | 0.40 | 1.60 |
| Nonmaternal older sisters | 0.41 | 0.38 | 1.07 | .283 | 1.51 | 0.71 | 3.21 |

Note: Estimates represent the log odds using tops as a referential value.

Table S6: Novel parameterization of AERO preferences in gay men (covariates as preregistered)

|  |  |  |  |  |  | 95% CI | |
| --- | --- | --- | --- | --- | --- | --- | --- |
|  | *b* | *SE* | *Z* | *p* | OR | Lower | Upper |
| Intercept | 0.89 | 0.20 | 4.54 | < .001 | 2.44 | 1.66 | 3.59 |
| Sibship size | -0.25 | 0.24 | -1.04 | .298 | 0.78 | 0.48 | 1.25 |
| Maternal older siblings | -0.10 | 0.26 | -0.40 | .692 | 0.90 | 0.54 | 1.51 |
| Maternal older brothers | 0.13 | 0.23 | 0.54 | .591 | 1.13 | 0.72 | 1.79 |
| Maternal younger brothers | 0.00 | 0.30 | 0.01 | .994 | 1.00 | 0.56 | 1.80 |

Note: Estimates represent the log odds using tops as a referential value.

Table S7: Fraternal birth order effect in women (covariates as preregistered)

|  |  |  | |  |  |  |  |  |  |  |  | 95% CI | |
| --- | --- | --- | --- | --- | --- | --- | --- | --- | --- | --- | --- | --- | --- |
|  | *b* | | *SE* | | | *Z* | | *p* | | OR | | Lower | Upper |
| Intercept | 1.67 | | 0.42 | | | 3.94 | | < .001 | | 5.32 | | 2.31 | 12.24 |
| Maternal younger brothers | 0.05 | | 0.14 | | | 0.36 | | .723 | | 1.05 | | 0.79 | 1.39 |
| Maternal older brothers | 0.38 | | 0.16 | | | 2.37 | | .018 | | 1.46 | | 1.07 | 2.01 |
| Maternal younger sisters | 0.28 | | 0.16 | | | 1.72 | | .085 | | 1.32 | | 0.96 | 1.81 |
| Maternal older sisters | 0.07 | | 0.16 | | | 0.46 | | .644 | | 1.08 | | 0.79 | 1.47 |
| Nonmaternal younger brothers | 0.42 | | 0.20 | | | 2.05 | | .040 | | 1.52 | | 1.02 | 2.27 |
| Nonmaternal older brothers | 0.23 | | 0.22 | | | 1.07 | | .283 | | 1.26 | | 0.83 | 1.92 |
| Nonmaternal younger sisters | -0.31 | | 0.23 | | | -1.35 | | .176 | | 0.73 | | 0.46 | 1.15 |
| Nonmaternal older sisters | 0.27 | | 0.21 | | | 1.24 | | .213 | | 1.30 | | 0.86 | 1.98 |
| Age | -0.05 | | 0.01 | | | -4.59 | | < .001 | | 0.95 | | 0.93 | 0.97 |
| Importance of religious faith | -0.02 | | 0.00 | | | -5.57 | | < .001 | | 0.98 | | 0.97 | 0.99 |
| Population size of place of residence (childhood)^a^  2–1 | -0.24 | | 0.33 | | | -0.72 | | .470 | | 0.79 | | 0.41 | 1.50 |
| 3–1 | -0.11 | | 0.28 | | | -0.40 | | .693 | | 0.89 | | 0.51 | 1.55 |
| 4–1 | -0.37 | | 0.30 | | | -1.23 | | .219 | | 0.69 | | 0.38 | 1.25 |
| 5–1 | -0.53 | | 0.34 | | | -1.57 | | .115 | | 0.59 | | 0.30 | 1.14 |

Note: Estimates represent the log odds using heterosexuals as a referential value.

^a^ Rows represent a comparison against a baseline (“1”), which is the option with the lowest number of inhabitants. See description of relevant variables in Methods.

Table S8: Novel parameterization of fraternal birth order effect in women (covariates as preregistered)

|  |  |  |  |  |  | 95% CI | |
| --- | --- | --- | --- | --- | --- | --- | --- |
|  | *b* | *SE* | *Z* | *p* | OR | Lower | Upper |
| Intercept | 1.83 | 0.42 | 4.36 | < .001 | 6.24 | 2.74 | 14.20 |
| Sibship size | 0.26 | 0.16 | 1.63 | .104 | 1.29 | 0.95 | 1.76 |
| Maternal older siblings | -0.19 | 0.21 | -0.89 | .371 | 0.83 | 0.55 | 1.25 |
| Maternal older brothers | 0.34 | 0.21 | 1.59 | .112 | 1.40 | 0.92 | 2.13 |
| Maternal younger brothers | -0.18 | 0.20 | -0.91 | .364 | 0.83 | 0.56 | 1.24 |
| Age | -0.06 | 0.01 | -4.84 | < .001 | 0.95 | 0.92 | 0.97 |
| Importance of religious faith | -0.02 | 0.00 | -5.51 | < .001 | 0.98 | 0.97 | 0.99 |
| Population size of place of residence (childhood)^a^ |  |  |  |  |  |  |  |
| 2–1 | -0.22 | 0.32 | -0.69 | .488 | 0.80 | 0.42 | 1.51 |
| 3–1 | -0.10 | 0.28 | -0.37 | .709 | 0.90 | 0.52 | 1.56 |
| 4–1 | -0.37 | 0.30 | -1.23 | .218 | 0.69 | 0.38 | 1.24 |
| 5–1 | -0.53 | 0.33 | -1.61 | .108 | 0.59 | 0.31 | 1.12 |

Note: Estimates represent the log odds using heterosexuals as a referential value.

Table S9: AERO behavior in straight men (covariates as preregistered)

|  | | | | | | 95% CI | |
| --- | --- | --- | --- | --- | --- | --- | --- |
|  | *b* | *SE* | *Z* | *p* | OR | Lower | Upper |
| Intercept | -2.09 | 0.30 | -6.86 | < .001 | 0.12 | 0.07 | 0.22 |
| Maternal younger brothers | 0.14 | 0.27 | 0.51 | .608 | 1.15 | 0.67 | 1.97 |
| Maternal younger sisters | 0.38 | 0.25 | 1.51 | .131 | 1.46 | 0.89 | 2.39 |
| Maternal older brothers | -0.43 | 0.42 | -1.04 | .300 | 0.65 | 0.29 | 1.47 |
| Maternal older sisters | 0.49 | 0.28 | 1.76 | .078 | 1.63 | 0.95 | 2.80 |
| Nonmaternal younger brothers | -0.08 | 0.41 | -0.19 | .848 | 0.92 | 0.41 | 2.07 |
| Nonmaternal younger sisters | -0.23 | 0.45 | -0.51 | .611 | 0.80 | 0.33 | 1.91 |
| Nonmaternal older brothers | 0.35 | 1.06 | 0.33 | .738 | 1.42 | 0.18 | 11.29 |
| Nonmaternal older sisters | -15.45 | 971.02 | -0.02 | .987 | 0.00 | 0.00 | Inf |

Note: Estimates represent the log odds using tops as a referential value.

Table S10: Novel parameterization of AERO behavior in straight men (covariates as preregistered)

|  |  |  |  |  |  | 95% CI | |
| --- | --- | --- | --- | --- | --- | --- | --- |
|  | *b* | *SE* | *Z* | *p* | OR | Lower | Upper |
| Intercept | -2.16 | 0.29 | -7.43 | < .001 | 0.12 | 0.07 | 0.20 |
| Sibship size | 0.37 | 0.25 | 1.50 | .134 | 1.45 | 0.89 | 2.37 |
| Maternal older siblings | 0.13 | 0.35 | 0.37 | .711 | 1.14 | 0.57 | 2.26 |
| Maternal older brothers | -0.92 | 0.53 | -1.74 | .082 | 0.40 | 0.14 | 1.13 |
| Maternal younger brothers | -0.29 | 0.38 | -0.76 | .448 | 0.75 | 0.35 | 1.58 |

Note: Estimates represent the log odds using tops as a referential value.

Table S11: AERO preferences in straight men (covariates as preregistered)

|  | | | | | | 95% CI | |
| --- | --- | --- | --- | --- | --- | --- | --- |
|  | *b* | *SE* | *Z* | *p* | OR | Lower | Upper |
| Intercept | -1.48 | 0.19 | -7.74 | < .001 | 0.23 | 0.16 | 0.33 |
| Maternal younger brothers | -0.18 | 0.19 | -0.98 | .325 | 0.83 | 0.58 | 1.20 |
| Maternal younger sisters | 0.42 | 0.17 | 2.52 | .012 | 1.52 | 1.10 | 2.10 |
| Maternal older brothers | 0.10 | 0.19 | 0.51 | .608 | 1.10 | 0.76 | 1.62 |
| Maternal older sisters | 0.24 | 0.17 | 1.41 | .159 | 1.27 | 0.91 | 1.78 |
| Nonmaternal younger brothers | -0.19 | 0.30 | -0.63 | .531 | 0.83 | 0.46 | 1.50 |
| Nonmaternal younger sisters | 0.60 | 0.29 | 2.05 | .040 | 1.82 | 1.03 | 3.23 |
| Nonmaternal older brothers | -0.05 | 0.45 | -0.10 | .919 | 0.95 | 0.39 | 2.31 |
| Nonmaternal older sisters | 0.25 | 0.40 | 0.63 | .526 | 1.29 | 0.59 | 2.81 |

Note: Estimates represent the log odds using tops as a referential value.

Table S12: Novel parameterization of AERO preferences in straight men (covariates as preregistered)

|  |  |  |  |  |  | 95% CI | |
| --- | --- | --- | --- | --- | --- | --- | --- |
|  | *b* | *SE* | *Z* | *p* | OR | Lower | Upper |
| Intercept | -1.41 | 0.18 | -7.66 | < .001 | 0.24 | 0.17 | 0.35 |
| Sibship size | 0.42 | 0.16 | 2.58 | .010 | 1.52 | 1.11 | 2.10 |
| Maternal older siblings | -0.19 | 0.21 | -0.88 | .377 | 0.83 | 0.54 | 1.26 |
| Maternal older brothers | -0.17 | 0.26 | -0.66 | .506 | 0.84 | 0.51 | 1.39 |
| Maternal younger brothers | -0.59 | 0.24 | -2.42 | .015 | 0.56 | 0.35 | 0.89 |

Note: Estimates represent the log odds using tops as a referential value.

Table S13: Proportions of brothers in analytical subsamples

|  | Older bros. | Older sis. | Older sibs. Total | % of bros. | *p* | Younger bros. | Younger sis. | Younger sibs. Total | % of bros. | *p* |
| --- | --- | --- | --- | --- | --- | --- | --- | --- | --- | --- |
| Gay men | 283 | 235 | 518 | 0.55 | .154 | 245 | 183 | 428 | 0.57 | .017 |
| Straight men | 254 | 260 | 514 | 0.49 | .345 | 361 | 318 | 679 | 0.53 | .385 |
| Top gay men  (AERO behavior) | 58 | 39 | 97 | 0.60 | .102 | 58 | 35 | 93 | 0.62 | .036 |
| Bottom straight men (AERO behavior) | 52 | 47 | 99 | 0.53 | .838 | 55 | 46 | 101 | 0.54 | .552 |
| Top gay men  (AERO preferences) | 63 | 55 | 118 | 0.53 | .681 | 41 | 30 | 71 | 0.58 | .292 |
| Bottom gay men (AERO preferences) | 84 | 62 | 146 | 0.58 | .145 | 59 | 46 | 105 | 0.56 | .336 |
| Top straight men  (AERO behavior) | 52 | 58 | 110 | 0.47 | .375 | 91 | 77 | 168 | 0.54 | .489 |
| Bottom straight men (AERO behavior) | 5 | 15 | 20 | 0.25 | .018 | 16 | 18 | 34 | 0.47 | .604 |
| Top straight men  (AERO preferences) | 92 | 100 | 192 | 0.48 | .321 | 157 | 122 | 279 | 0.56 | .111 |
| Bottom straight men (AERO preferences) | 31 | 39 | 70 | 0.44 | .227 | 38 | 55 | 93 | 0.41 | < .001 |
| Lesbian women | 98 | 85 | 183 | 0.54 | .579 | 92 | 94 | 186 | 0.49 | .578 |
| Straight women | 94 | 99 | 193 | 0.49 | .437 | 117 | 105 | 222 | 0.53 | .720 |

^a^ Expected value was 0.515.

Bros. – brothers; sis. – sisters; sibs. – siblings; % – proportion.

Table S14: AERO behavior in straight men

|  |  |  |  |  |  | 95% CI | |
| --- | --- | --- | --- | --- | --- | --- | --- |
|  | *b* | *SE* | *Z* | *p* | OR | Lower | Upper |
| Intercept | -3.35 | 1.63 | -2.05 | .040 | 0.04 | 0.00 | 0.86 |
| Maternal younger brothers | 0.15 | 0.28 | 0.54 | .589 | 1.16 | 0.68 | 1.99 |
| Maternal younger sisters | 0.41 | 0.26 | 1.61 | .108 | 1.51 | 0.91 | 2.50 |
| Maternal older brothers | -0.44 | 0.42 | -1.03 | .303 | 0.65 | 0.28 | 1.48 |
| Maternal older sisters | 0.43 | 0.29 | 1.48 | .138 | 1.54 | 0.87 | 2.72 |
| Nonmaternal younger brothers | -0.03 | 0.40 | -0.08 | .940 | 0.97 | 0.45 | 2.11 |
| Nonmaternal younger sisters | -0.19 | 0.44 | -0.44 | .662 | 0.82 | 0.35 | 1.96 |
| Nonmaternal older brothers | 0.40 | 1.07 | 0.37 | .710 | 1.49 | 0.18 | 12.04 |
| Nonmaternal older sisters | -15.33 | 974.70 | -0.02 | .987 | 0.00 | 0.00 | Inf |
| Age | 0.02 | 0.02 | 0.95 | .340 | 1.02 | 0.98 | 1.05 |
| Mother’s age at target’s birth | 0.02 | 0.05 | 0.44 | .659 | 1.02 | 0.92 | 1.14 |

Note: Estimates represent the log odds using tops as a referential value.

Table S15: Novel parameterization of AERO behavior in straight men

|  |  |  |  |  |  | 95% CI | |
| --- | --- | --- | --- | --- | --- | --- | --- |
|  | *b* | *SE* | *Z* | *p* | OR | Lower | Upper |
| Intercept | -3.44 | 1.58 | -2.18 | .029 | 0.03 | 0.00 | 0.71 |
| Sibship size | 0.41 | 0.25 | 1.60 | .110 | 1.50 | 0.91 | 2.47 |
| Maternal older siblings | 0.04 | 0.37 | 0.12 | .905 | 1.05 | 0.51 | 2.16 |
| Maternal older brothers | -0.85 | 0.53 | -1.59 | .112 | 0.43 | 0.15 | 1.22 |
| Maternal younger brothers | -0.29 | 0.38 | -0.77 | .441 | 0.75 | 0.35 | 1.58 |
| Age | 0.02 | 0.02 | 1.22 | .223 | 1.02 | 0.99 | 1.06 |
| Mother’s age at target’s birth | 0.02 | 0.05 | 0.37 | .714 | 1.02 | 0.92 | 1.13 |

Note: Estimates represent the log odds using tops as a referential value.

Table S16: AERO preferences in straight men

|  |  |  |  |  |  | 95% CI | |
| --- | --- | --- | --- | --- | --- | --- | --- |
|  | *b* | *SE* | *Z* | *p* | OR | Lower | Upper |
| Intercept | -2.74 | 0.93 | -2.95 | .003 | 0.06 | 0.01 | 0.40 |
| Maternal younger brothers | -0.18 | 0.19 | -0.93 | .351 | 0.84 | 0.58 | 1.21 |
| Maternal younger sisters | 0.42 | 0.17 | 2.52 | .012 | 1.53 | 1.10 | 2.13 |
| Maternal older brothers | 0.11 | 0.20 | 0.56 | .573 | 1.12 | 0.76 | 1.66 |
| Maternal older sisters | 0.19 | 0.18 | 1.05 | .292 | 1.21 | 0.85 | 1.71 |
| Nonmaternal younger brothers | -0.17 | 0.30 | -0.55 | .581 | 0.85 | 0.47 | 1.53 |
| Nonmaternal younger sisters | 0.62 | 0.29 | 2.11 | .035 | 1.86 | 1.05 | 3.32 |
| Nonmaternal older brothers | -0.04 | 0.45 | -0.09 | .927 | 0.96 | 0.40 | 2.32 |
| Nonmaternal older sisters | 0.27 | 0.40 | 0.67 | .501 | 1.31 | 0.59 | 2.90 |
| Age | 0.02 | 0.01 | 1.84 | .065 | 1.02 | 1.00 | 1.04 |
| Mother’s age at childbirth | 0.02 | 0.03 | 0.79 | .427 | 1.02 | 0.97 | 1.09 |

Note: Estimates represent the log odds using tops as a referential value.

Table S17: Novel parameterization of AERO preferences in straight men

|  |  |  |  |  |  | 95% CI | |
| --- | --- | --- | --- | --- | --- | --- | --- |
|  | *b* | *SE* | *Z* | *p* | OR | Lower | Upper |
| Intercept | -2.54 | 0.92 | -2.76 | .006 | 0.08 | 0.01 | 0.48 |
| Sibship size | 0.43 | 0.17 | 2.56 | .010 | 1.53 | 1.10 | 2.12 |
| Maternal older siblings | -0.24 | 0.23 | -1.05 | .292 | 0.79 | 0.50 | 1.23 |
| Maternal older brothers | -0.11 | 0.26 | -0.41 | .680 | 0.90 | 0.54 | 1.50 |
| Maternal younger brothers | -0.58 | 0.24 | -2.39 | .017 | 0.56 | 0.35 | 0.90 |
| Age | 0.02 | 0.01 | 1.79 | .074 | 1.02 | 1.00 | 1.04 |
| Mother’s age at target’s birth | 0.02 | 0.03 | 0.67 | .502 | 1.02 | 0.96 | 1.08 |

Note: Estimates represent the log odds using tops as a referential value.
